# Supplementary material for: Dysregulation of the leukocyte signaling landscape during acute COVID-19
Source: PLoS One. 2022 Apr 14;17(4):e0264979. doi: 10.1371/journal.pone.0264979 (PMC9009616; doi:10.1371/journal.pone.0264979)
Supplement: S1 Table — (PDF) [file pone.0264979.s001.pdf]

Label	Target	Clone	Manufacturer	Titer
Surface Staining Panel				
089Y	CD45	HI30	Fluidigm	1x
113In	CD66b	B1.1*	BD Biosciences	0.312 µg/ml
115In	CD3	UCHT1	Biolegend	0.544 µg/ml
141Pr	CD33	WM53*	Biolegend	0.312 µg/ml
142Nd	CD19	HIB19	Fluidigm	0.062x
143Nd	CD127	A019D5	Fluidigm	1x
145Nd	CD4	RPA-T4	Fluidigm	0.125x
147Sm	CD11c	Bu15	Fluidigm	0.125x
148Nd	CD14	RMO52	Fluidigm	0.016x
149Sm	CD45RO	UCHL1	Fluidigm	0.25x
151Eu	CD123	6H6	Fluidigm	0.109x
155Gd	CD27	L128	Fluidigm	0.062x
160Gd	CD69	FN50	BioLegend	0.625x
161Dy	CD11b (Mac-1)	ICRF44*	Biolegend	0.312 µg/ml
163Dy	CD235ab	HIR2	Fluidigm	0.109x
163Dy	CD61	VI-PL2*	Biolegend	0.033 µg/ml
168Er	CD8	SK1	Fluidigm	0.031x
169Tm	CD25	2A3	Fluidigm	0.062x
170Er	Siglec 8	7C9*	Biolegend	2.5 µg/ml
172Yb	CD15 (SSEA-1)	W6D3	Fluidigm	0.125x
173Yb	HLA-DR	L243	Fluidigm	0.031x
175Lu	PD-1	EH12.2H7	Fluidigm	0.062x
176Yb	CD56	NCAM16.2	Fluidigm	0.109x
209Bi	CD16	3G8	Fluidigm	0.062x
Intracellular Staining Panel				
144Nd	pPLCG2	K86-689.37	Fluidigm	0.33x
150Nd	pSTAT5	47	Fluidigm	1x
152Sm	pAKT	D9E	Fluidigm	0.33x
153Eu	pSTAT1	58D6	Fluidigm	0.33x
154Sm	pBtk/Itk	24a/BTK	BD Biosciences	0.5 µg/ml
156Gd	p38 [T180/Y182]	D3F9	Fluidigm	0.109x
158Gd	pSTAT3	4/P-Stat3	Fluidigm	1x
159Tb	pMAPKAPK2	27B7	Fluidigm	0.109x
162Dy	pLCK	4/LCK-Y505	Fluidigm	0.33x
164Dy	IkBa	L35A5	Fluidigm	0.33x
165Ho	pCREB	87G3	Fluidigm	0.033x
166Er	pNFKB	K10-895.12.50	Fluidigm	0.054x
167Er	pERK	D13.14.4E	Fluidigm	0.1x
171Yb	pZAP70	17a	Fluidigm	1x

Supplemental Table 1. Antibody panels for surface and intracellular staining.
