# Supplementary material for: Dysregulation of the leukocyte signaling landscape during acute COVID-19
Source: PLoS One. 2022 Apr 14;17(4):e0264979. doi: 10.1371/journal.pone.0264979 (PMC9009616; doi:10.1371/journal.pone.0264979)
Supplement: S2 Table — (PDF) [file pone.0264979.s002.pdf]

	Healthy Control	Moderate COVID19	Severe COVID19
n	18	20	43
Age (years, mean+/-SD)	52.5+/-10.8	55.6+/-14.6	60.2+/-18.4
Female (n (%))	12 (66.7)	8 (40.0)	21 (48.8)
Race/Ethnicity (n(%))			
White	11 (61.1)	3 (15.0)	11 (25.6)
Black	5 (27.8)	17 (85.0)	32 (74.4)
Asian	1 (5.6)	0	0
Hispanic	1 (5.6)	0	0
Length of Stay (days, mean+/-SD)	[redacted]	5.7+/-4.0	17.9+/-10.1
Days of Symptoms at hospitalization (mean+/-SD)	[redacted]	9.0+/-11.2	6.7+/-6.6
Hospital Day of Sample (mean+/-SD)	[redacted]	0.5+/-1.1	2.1+/-2.9
ICU (n (%))	[redacted]	0	40 (93.0)
Mechanical Ventilation (n(%))	[redacted]	0	25 (58.1)
Mortality (n,(%))	[redacted]	0	16 (37)
