# Supplementary material for: Dysregulation of the leukocyte signaling landscape during acute COVID-19
Source: PLoS One. 2022 Apr 14;17(4):e0264979. doi: 10.1371/journal.pone.0264979 (PMC9009616; doi:10.1371/journal.pone.0264979)
Supplement: S3 Table — (PDF) [file pone.0264979.s003.pdf]

| | | Moderate COVI | |
|---|---|---|---|
| | Reference Range | Mean | Std. Dev |
| ALANINE AMINOTRANSFERASE (Units/L) | 7-55 | 52.89 | 75.19 |
| ALBUMIN (g/dL) | 3.5-5 | 3.39 | 0.96 |
| ALKALINE PHOSPHATASE (Units/L) | 40-130 | 79.37 | 53.68 |
| ANION GAP (mmol/L) | 2-15 | 10.17 | 3.56 |
| ASPARTATE AMINOTRANSFERASE (Units/L) | 10-50 | 60.11 | 65.84 |
| BASOPHIL ABSOLUTE (K/cumm) | 0-0.1 | 0.03 | 0.06 |
| BASOPHIL PERCENT (%) | Not reported | 0.38 | 0.28 |
| BILIRUBIN, TOTAL (mg/dL) | 0.1-1.2 | 0.57 | 0.33 |
| CALCIUM, TOTAL (mg/dL) | 8.5-10.3 | 8.51 | 1.94 |
| CHLORIDE (mmol/L) | 97-110 | 95.04 | 21.26 |
| CO2, TOTAL (mmol/L) | 22-32 | 25.17 | 6.12 |
| CREATININE (mg/dL) | 0.8-1.3 | 1.52 | 1.42 |
| EOSINOPHIL PERCENT (%) | Not reported | 0.81 | 1.21 |
| GLUCOSE (mg/dL) | 70-199 | 129.17 | 65.37 |
| HEMATOCRIT (%) | 38.9-50.3 | 34.85 | 10.04 |
| HEMOGLOBIN (g/dL) | 13-17.5 | 11.22 | 3.38 |
| IMMATURE GRANULOCYTE ABSOLUTE (K/cumm) | 0-0.1 | 0.09 | 0.23 |
| IMMATURE GRANULOCYTE PERCENT (%) | Not reported | 0.81 | 0.70 |
| LYMPHOCYTE ABSOLUTE (K/cumm) | 0.8-3.3 | 1.23 | 0.62 |
| LYMPHOCYTE PERCENT (%) | Not reported | 18.41 | 9.43 |
| MEAN CELLULAR HEMOGLOBIN (pg) | 27.1-33.3 | 26.70 | 6.60 |
| MEAN CELLULAR HEMOGLOBIN CONCENTRATION (g/ | 32.3-35.7 | 30.73 | 6.97 |
| MEAN CELLULAR VOLUME (fL) | 81.3-96.4 | 83.03 | 19.74 |
| MEAN PLATELET VOLUME (fL) | 9.1-12.3 | 10.47 | 2.62 |
| MONOCYTE ABSOLUTE (K/cumm) | 0.2-0.8 | 0.70 | 0.65 |
| MONOCYTE PERCENT (%) | Not reported | 9.01 | 5.25 |
| NEUTROPHIL ABSOLUTE (K/cumm) | 1.7-6.5 | 5.78 | 7.06 |
| NEUTROPHIL PERCENT (%) | Not reported | 64.96 | 18.98 |
| NUCLEATED RBC ABS, AUTO (K/cumm) | 0-0.01 | 0.00 | 0.01 |
| PLATELET (K/cumm) | 150-400 | 198.00 | 68.42 |
| POTASSIUM, PLASMA (mmol/L) | 3.3-4.9 | 3.81 | 0.99 |
| PROTEIN, TOTAL, PLASMA (g/dL) | 6.5-8.5 | 7.22 | 1.86 |
| RED BLOOD CELL (M/cumm) | 4.3-5.8 | 4.08 | 1.34 |
| RED CELL DISTRIBUTION WIDTH CV (%) | 11.1-14.9 | 14.31 | 3.58 |
| RED CELL DISTRIBUTION WIDTH SD (fL) | 35.7-48.1 | 44.97 | 11.89 |
| SODIUM (mmol/L) | 135-145 | 130.39 | 28.77 |
| UREA NITROGEN SERUM (mg/dL) | 8-25 | 22.00 | 20.13 |
| WHITE BLOOD CELL COUNT (K/cumm) | 3.8-9.9 | 7.24 | 7.21 |
| MAGNESIUM (mg/dL) | 1.4-2.5 | 1.76 | 0.67 |
| D-DIMER (ng/mL FEU) | <=499 | 1471.25 | 1485.83 |
| FERRITIN (ng/mL) | 30-400 | 724.00 | 832.48 |
| INTERNATIONAL NORMALIZED RATIO () | 0.9-1.1 | 1.15 | 0.49 |
| CREATINE KINASE, TOTAL (Units/L) | 30-200 | 331.80 | 514.94 |
| C REACTIVE PROTEIN (mg/L) | <=10 | 77.50 | 63.81 |
| LACTATE DEHYDROGENASE, TOTAL (Units/L) | 100-250 | 294.60 | 142.38 |

| | | | |
|---|---|---|---|
| ERYTHROCYTE SEDIMENTATION RATE (mm/hr) | 1-20 | 41.20 | 40.76 |

| D19 | | Severe COVID19 | | Moderate vs. Severe |
| --- | --- | --- | --- | --- |
| N | Mean | Std. Dev | N | p-value (MannU) |
| 19.00 | 66.81 | 83.14 | 26.00 | 5.42E-01 |
| 19.00 | 3.02 | 0.67 | 26.00 | 8.21E-03 |
| 19.00 | 113.35 | 146.11 | 26.00 | 3.71E-01 |
| 23.00 | 10.10 | 3.20 | 30.00 | 4.83E-01 |
| 19.00 | 151.81 | 399.47 | 26.00 | 3.28E-01 |
| 18.00 | 0.02 | 0.05 | 26.00 | 5.09E-01 |
| 18.00 | 0.22 | 0.16 | 26.00 | 2.05E-02 |
| 18.00 | 0.74 | 0.99 | 25.00 | 5.87E-01 |
| 23.00 | 8.61 | 0.80 | 30.00 | 3.25E-03 |
| 23.00 | 104.77 | 7.50 | 30.00 | 2.61E-03 |
| 23.00 | 24.63 | 4.85 | 30.00 | 2.43E-01 |
| 23.00 | 1.70 | 1.53 | 40.00 | 8.14E-01 |
| 18.00 | 0.56 | 0.96 | 28.00 | 3.97E-01 |
| 23.00 | 144.30 | 46.85 | 30.00 | 1.54E-01 |
| 23.00 | 33.49 | 6.36 | 30.00 | 8.66E-02 |
| 23.00 | 10.77 | 2.07 | 30.00 | 1.24E-01 |
| 19.00 | 0.16 | 0.32 | 28.00 | 2.33E-01 |
| 17.00 | 1.29 | 1.91 | 26.00 | 9.90E-01 |
| 19.00 | 1.09 | 0.40 | 28.00 | 1.15E-01 |
| 19.00 | 13.43 | 8.82 | 28.00 | 2.44E-02 |
| 23.00 | 28.15 | 2.87 | 30.00 | 8.75E-01 |
| 23.00 | 32.17 | 1.49 | 30.00 | 6.77E-01 |
| 23.00 | 87.48 | 7.59 | 30.00 | 9.48E-01 |
| 23.00 | 10.49 | 1.05 | 30.00 | 1.85E-01 |
| 19.00 | 0.66 | 0.41 | 28.00 | 9.93E-01 |
| 19.00 | 6.56 | 4.30 | 28.00 | 1.70E-02 |
| 19.00 | 9.39 | 6.33 | 28.00 | 3.90E-03 |
| 19.00 | 78.04 | 10.76 | 28.00 | 9.64E-03 |
| 23.00 | 0.02 | 0.05 | 30.00 | 1.05E-01 |
| 23.00 | 268.53 | 156.49 | 40.00 | 4.23E-01 |
| 23.00 | 4.30 | 0.61 | 30.00 | 4.60E-02 |
| 19.00 | 6.98 | 1.05 | 26.00 | 2.96E-02 |
| 23.00 | 3.85 | 0.80 | 30.00 | 1.22E-01 |
| 23.00 | 15.62 | 2.40 | 30.00 | 3.74E-01 |
| 23.00 | 49.37 | 6.52 | 30.00 | 2.21E-01 |
| 23.00 | 139.50 | 7.29 | 30.00 | 1.96E-01 |
| 23.00 | 28.68 | 21.05 | 40.00 | 1.02E-01 |
| 23.00 | 11.61 | 6.43 | 40.00 | 8.16E-04 |
| 14.00 | 2.17 | 0.26 | 26.00 | 9.54E-02 |
| 16.00 | 8835.12 | 14601.13 | 33.00 | 2.13E-02 |
| 12.00 | 1585.82 | 2169.97 | 28.00 | 1.19E-01 |
| 13.00 | 1.36 | 0.24 | 28.00 | 2.01E-02 |
| 5.00 | 1438.38 | 1270.83 | 8.00 | 8.94E-02 |
| 15.00 | 172.62 | 125.42 | 31.00 | 2.26E-02 |
| 10.00 | 597.76 | 365.50 | 17.00 | 1.90E-02 |

| | | | | |
|---|---|---|---|---|
| 5.00 | 58.13 | 38.28 | 8.00 | 6.09E-01 |
