# Supplementary material for: Dysregulation of the leukocyte signaling landscape during acute COVID-19
Source: PLoS One. 2022 Apr 14;17(4):e0264979. doi: 10.1371/journal.pone.0264979 (PMC9009616; doi:10.1371/journal.pone.0264979)
Supplement: S4 Table — (PDF) [file pone.0264979.s004.pdf]

Feature	Healthy Donor*	Moderate COVID19*	Severe COVID19*	P value	q value	Discovery? (1% FDR)
PMN_pLCK	0.95+/-0.33	1+/-0.12	0.93+/-0.09	0.07	0.07	No
PMN_IkBa	0.98+/-0.08	1+/-0.1	0.99+/-0.07	0.94	0.58	No
PMN_pNKFb	1.05+/-0.19	1.06+/-0.1	1.11+/-0.2	0.18	0.15	No
PMN_pERK	1+/-0.01	1.01+/-0.01	1.04+/-0.05	0.00	0.00	Yes
PMN_pSTAT1	1+/-0.04	1.03+/-0.06	1.07+/-0.11	0.00	0.00	Yes
PMN_p38	1.24+/-0.47	1.03+/-0.06	1.04+/-0.09	0.47	0.31	No
PMN_pSTAT3	0.91+/-0.15	1.44+/-1.11	1.52+/-0.9	0.06	0.06	No
PMN_pCREB	1.04+/-0.13	0.99+/-0.06	1.03+/-0.17	0.33	0.24	No
PMN_PLCG2	1.01+/-0.01	1+/-0.02	1.01+/-0.02	0.26	0.20	No
PMN_pSTAT5	1.03+/-0.34	1.04+/-0.27	1.11+/-0.27	0.24	0.19	No
PMN_pAKT	0.99+/-0.01	1+/-0.02	1.01+/-0.02	0.02	0.02	No
PMN_pBtk/Itk	0.65+/-0.3	0.91+/-0.31	0.91+/-0.25	0.02	0.02	No
PMN_pMAPKAP2	1+/-0.03	1.02+/-0.04	1.07+/-0.17	0.15	0.13	No
PMN_pZAP70	1+/-0.02	1.01+/-0.03	1+/-0.03	0.99	0.61	No
PMN_pSTAT4	1.16+/-0.52	1.09+/-0.29	1.14+/-0.3	0.46	0.31	No
Mono_pLCK	1.12+/-0.11	1.05+/-0.1	0.98+/-0.07	0.00	0.00	Yes
Mono_IkBa	1.01+/-0.06	1.04+/-0.1	1.02+/-0.19	0.28	0.21	No
Mono_pNKFb	1.14+/-0.55	1.13+/-0.17	1.26+/-0.37	0.12	0.11	No
Mono_pERK	1+/-0.01	1.04+/-0.04	1.11+/-0.13	0.00	0.00	Yes
Mono_pSTAT1	1.04+/-0.07	1.08+/-0.12	1.21+/-0.32	0.05	0.06	No
Mono_p38	1.11+/-0.39	1.18+/-0.28	1.27+/-0.47	0.17	0.14	No
Mono_pSTAT3	1.31+/-0.39	2.3+/-1.37	3.24+/-2.17	0.00	0.00	Yes
Mono_pCREB	1.02+/-0.11	1.22+/-0.16	1.54+/-0.74	0.00	0.00	Yes
Mono_PLCG2	1.03+/-0.05	1+/-0.01	1.03+/-0.06	0.70	0.45	No
Mono_pSTAT5	1.44+/-0.95	0.99+/-0.25	1.31+/-0.61	0.24	0.19	No
Mono_pAKT	1.02+/-0.05	1+/-0.02	1.02+/-0.05	0.59	0.38	No
Mono_pBtk/Itk	0.87+/-0.3	0.95+/-0.07	1.01+/-0.2	0.08	0.08	No
Mono_pMAPKAP2	0.99+/-0.06	1.19+/-0.25	1.66+/-1.11	0.00	0.00	Yes
Mono_pZAP70	1.01+/-0.04	1+/-0.03	1.02+/-0.06	0.20	0.16	No
Mono_pSTAT4	1.01+/-0.35	0.95+/-0.27	1.08+/-0.26	0.09	0.09	No
CD4_pLCK	1.01+/-0.01	1.01+/-0.01	1.01+/-0.02	0.38	0.27	No
CD4_IkBa	1.01+/-0.04	1.02+/-0.09	1.14+/-0.26	0.00	0.01	Yes
CD4_pNKFb	1.25+/-0.48	1.03+/-0.22	1.02+/-0.29	0.54	0.36	No
CD4_pERK	1+/-0.01	1+/-0.01	1+/-0.01	0.08	0.08	No
CD4_pSTAT1	1.01+/-0.01	1.04+/-0.05	1.14+/-0.16	0.00	0.00	Yes
CD4_p38	1.1+/-0.3	1.06+/-0.12	1+/-0.1	0.45	0.31	No
CD4_pSTAT3	1.3+/-0.34	3.97+/-2.68	7.7+/-3.64	0.00	0.00	Yes
CD4_pCREB	1.02+/-0.04	1.09+/-0.08	1.17+/-0.23	0.00	0.00	Yes
CD4_PLCG2	1.01+/-0.01	1.01+/-0.01	1.02+/-0.03	0.54	0.36	No
CD4_pSTAT5	1.02+/-0.15	0.93+/-0.27	1.06+/-0.3	0.09	0.08	No
CD4_pAKT	1.01+/-0.01	1.01+/-0.02	1.02+/-0.03	0.02	0.02	No
CD4_pBtk/Itk	0.83+/-0.16	0.97+/-0.05	1.01+/-0.11	0.00	0.00	Yes
CD4_pMAPKAP2	1.02+/-0.04	1.12+/-0.2	1.3+/-0.53	0.00	0.00	Yes
CD4_pZAP70	1.02+/-0.05	1.03+/-0.06	1.05+/-0.07	0.19	0.15	No
CD4_pSTAT4	0.99+/-0.2	1.11+/-0.23	1.17+/-0.2	0.01	0.01	No
CD8_pLCK	1.01+/-0.02	1.01+/-0.01	1.02+/-0.02	0.77	0.49	No
CD8_IkBa	1+/-0.04	1.02+/-0.09	1.09+/-0.18	0.01	0.01	Yes
CD8_pNKFb	1.51+/-0.7	1.13+/-0.2	1.17+/-0.35	0.42	0.30	No
CD8_pERK	1+/-0.03	0.99+/-0.03	1.01+/-0.03	0.02	0.02	No
CD8_pSTAT1	1.01+/-0.01	1.03+/-0.04	1.08+/-0.11	0.00	0.00	Yes
CD8_p38	1.18+/-0.43	1.11+/-0.2	1.01+/-0.12	0.26	0.20	No
CD8_pSTAT3	1.17+/-0.25	2.23+/-1.04	3.46+/-1.97	0.00	0.00	Yes
CD8_pCREB	1.05+/-0.04	1.11+/-0.1	1.23+/-0.29	0.00	0.00	Yes

CD8_PLCG2	1+/-0	1+/-0	1.01+/-0.02	0.00	0.00	Yes
CD8_pSTAT5	1.09+/-0.15	0.94+/-0.23	1.03+/-0.22	0.01	0.02	No
CD8_pAKT	1.01+/-0.01	1.01+/-0.01	1.02+/-0.03	0.26	0.20	No
CD8_pBtk/Itk	0.81+/-0.16	0.95+/-0.05	0.98+/-0.08	0.00	0.00	Yes
CD8_pMAPKAP2	1.02+/-0.04	1.1+/-0.18	1.25+/-0.45	0.00	0.00	Yes
CD8_pZAP70	1.01+/-0.04	1.02+/-0.07	1.03+/-0.06	0.14	0.12	No
CD8_pSTAT4	0.94+/-0.17	1.02+/-0.17	1.06+/-0.16	0.03	0.03	No
NK_pLCK	1.01+/-0.07	0.98+/-0.05	1.03+/-0.08	0.02	0.03	No
NK_IkBa	1.03+/-0.04	1.04+/-0.1	1.16+/-0.22	0.01	0.01	Yes
NK_pNKFb	1.32+/-0.65	1.02+/-0.17	0.96+/-0.26	0.13	0.11	No
NK_pERK	1+/-0.01	1+/-0.01	1.03+/-0.04	0.00	0.00	Yes
NK_pSTAT1	1.01+/-0.02	1.02+/-0.03	1.06+/-0.06	0.00	0.00	Yes
NK_p38	1.17+/-0.51	1.13+/-0.28	1.03+/-0.24	0.37	0.26	No
NK_pSTAT3	1.15+/-0.25	1.48+/-0.34	1.98+/-0.79	0.00	0.00	Yes
NK_pCREB	1.02+/-0.05	1.08+/-0.09	1.26+/-0.35	0.00	0.00	Yes
NK_PLCG2	1+/-0.01	1+/-0	1.02+/-0.05	0.09	0.08	No
NK_pSTAT5	1.16+/-0.21	1.06+/-0.25	1.4+/-0.65	0.07	0.07	No
NK_pAKT	1+/-0.01	1.01+/-0.01	1.03+/-0.04	0.00	0.00	Yes
NK_pBtk/Itk	0.83+/-0.17	0.95+/-0.04	1.02+/-0.14	0.00	0.00	Yes
NK_pMAPKAP2	1.02+/-0.03	1.07+/-0.07	1.26+/-0.39	0.00	0.00	Yes
NK_pZAP70	1+/-0.02	1+/-0.01	1.02+/-0.04	0.07	0.07	No
NK_pSTAT4	1.1+/-0.27	1.17+/-0.2	1.31+/-0.31	0.03	0.03	No
PMN_CD16	0.76+/-0.2	1+/-0.65	0.84+/-0.53	0.63	0.41	No
PMN_CD11b	0.96+/-0.42	0.93+/-0.25	0.72+/-0.23	0.01	0.01	Yes
PMN_CD66b	0.98+/-0.2	1.13+/-0.28	0.99+/-0.36	0.06	0.07	No
PMN_CD33	1.38+/-1.22	1.05+/-0.85	1.02+/-0.74	0.97	0.60	No
PMN_CD11c	1.24+/-0.52	1.86+/-1.14	1.41+/-0.73	0.10	0.09	No
PMN_CD15	1.2+/-0.33	0.85+/-0.28	0.81+/-0.36	0.00	0.00	Yes
PMN_HLA-DR	1.13+/-0.2	0.91+/-0.21	0.87+/-0.32	0.00	0.00	Yes
Mono_CD11b	1.18+/-0.23	0.97+/-0.28	0.77+/-0.23	0.00	0.00	Yes
Mono_CD14	0.88+/-0.1	0.86+/-0.19	0.66+/-0.23	0.00	0.00	Yes
Mono_CD33	1.45+/-0.7	1.01+/-0.31	0.86+/-0.32	0.00	0.00	Yes
Mono_HLA-DR	0.53+/-0.23	0.4+/-0.29	0.36+/-0.29	0.01	0.01	Yes
B cells	1.94+/-1.02	2.17+/-1.85	1.81+/-1.64	0.44	0.30	No
basophils	0.6+/-0.29	0.36+/-0.43	0.15+/-0.13	0.00	0.00	Yes
classical monocytes	5.81+/-1.7	3.84+/-3.14	2.82+/-2.63	0.00	0.00	Yes
pDC	0.12+/-0.1	0.07+/-0.1	0.04+/-0.04	0.00	0.00	Yes
NK cells	2.92+/-1.57	2.73+/-1.84	1.45+/-1.29	0.00	0.00	Yes
NKT cells	0.66+/-0.88	1.15+/-0.94	0.74+/-1.29	0.03	0.03	No
T cells	18.06+/-3.72	15.56+/-9.16	6.94+/-5.09	0.00	0.00	Yes
CD4 T cells	11.61+/-3.23	9.28+/-5.68	4.78+/-3.61	0.00	0.00	Yes
CD8 T cells	3.91+/-2.65	4.99+/-4.18	1.6+/-1.53	0.00	0.00	Yes
Neutrophils	65.37+/-4.18	72.38+/-13.17	81.89+/-11.26	0.00	0.00	Yes

Immunologic Features Defined by CyTOF. * data presented as mean+/-StDev. P values calculated by Kruskall-Wallis ANOVA. Q values calculated by Benjamini, Krieger and Yekutieli for False Discovery Rate (FDR) of 1%.
