# Supplementary material for: Dysregulation of the leukocyte signaling landscape during acute COVID-19
Source: PLoS One. 2022 Apr 14;17(4):e0264979. doi: 10.1371/journal.pone.0264979 (PMC9009616; doi:10.1371/journal.pone.0264979)
Supplement: S5 Table — (PDF) [file pone.0264979.s005.pdf]

Soluble Marker	Healthy Donor (n=18; Mean+/- SD)	Moderate COVID19 (n=15; Mean+/-SD)	Severe COVID19 (n=21; Mean+/-SD)
**IL-6**	2.31+/-1.99	32.79+/-52.3	249.78+/-226.05
**MCP-1**	445.87+/-156.23	805.73+/-1023.88	1225.79+/-916.06
**HGF**	40.76+/-23.07	213.33+/-373.2	1595.74+/-2208.43
**IL-1RA**	33.32+/-10.4	165.23+/-120.27	229.59+/-158.55
**IP-10**	29.14+/-36.49	108.02+/-80.71	228.79+/-193.5
IL-2R	47.09+/-36.7	149.08+/-217.33	167.1+/-161.18
**MIG**	28.31+/-30.23	50.6+/-56.72	138.61+/-101.41
**IL-8**	14.62+/-3.35	77.64+/-69.91	148.4+/-104.65
FGF-basic	12.23+/-13.46	10.42+/-11.38	15.02+/-14.16
IL-13	3.08+/-2.01	3.86+/-1.87	5.64+/-3.74
MIP-1b	61.18+/-38.38	100.14+/-84.9	112.86+/-94.99
IL-15	32.21+/-29.83	98+/-160.02	103.25+/-135.13
EGF	68.06+/-31.13	137.92+/-140.79	117.23+/-104.89
IL-1a	3.37+/-4.68	45.97+/-165.11	7.76+/-88.69
IFN-g	2.68+/-2.6	3.64+/-7.21	2.43+/-4.53
IFN-a	24.91+/-27	37.94+/-27.14	45.33+/-34.49
IL-3	18.63+/-19.2	46.75+/-76.94	36.6+/-54.91
IL-2	9.64+/-10.86	104.86+/-311.14	44.66+/-175.44
IL-4	3.27+/-3.25	7.54+/-16.36	6.87+/-10.46
IL-1b	0.76+/-0.57	3.93+/-7.92	3.17+/-4.97
G-CSF	25.79+/-18.03	21.66+/-27.13	23.42+/-24.79
RANTES	1148.9+/-985.82	1192.94+/-860.78	2154.6+/-2986.73
IL-17A	4.56+/-4.86	12.53+/-29.86	3.83+/-17.28
VEGF	2.6+/-3.07	18.54+/-30.42	5.5+/-17.51
IL-17F	77.84+/-82.63	702.56+/-2092.1	205.97+/-1148.7
IL-9	1.77+/-3.03	51.49+/-126.37	4.75+/-70.12
TNFa	2.21+/-5.19	31+/-99.88	3.05+/-54.02
IL-10	30.87+/-58.67	15.22+/-13.37	55.86+/-65.32
IL-12	46.64+/-22.84	108.85+/-165.34	61.2+/-93.65
EOTAXIN	98.61+/-66.09	79.26+/-105.11	57.74+/-74.32
MIP-1a	2.05+/-4.18	10.27+/-21.84	38.83+/-67.18
GM-CSF	2.8+/-6.2	4.42+/-10.04	2.57+/-6.5
IL-5	2.12+/-2.16	2.09+/-3.18	6.94+/-13.12
IL-7	20.29+/-23.55	6.05+/-4.38	21.58+/-29.78
IL-22	42.17+/-33.71	38.37+/-54.47	53.63+/-47.32

P value (Kruskal-Wallis)	q value (Benjamini, Krieger and Yekutieli)	Discovery? (1%FDR)	Hierarchial Clustering
3.25E-08	9.20E-07	**Yes**	Cluster A
1.97E-03	7.97E-03	**Yes**	Cluster A
1.09E-07	1.03E-06	**Yes**	Cluster A
2.21E-07	1.56E-06	**Yes**	Cluster A
7.67E-06	4.34E-05	**Yes**	Cluster A
8.55E-03	2.23E-02	No	Cluster A
3.83E-04	1.80E-03	**Yes**	Cluster A
6.84E-08	9.67E-07	**Yes**	Cluster A
1.79E-01	2.30E-01	No	Cluster B
1.04E-02	2.45E-02	No	Cluster B
2.20E-01	2.40E-01	No	Cluster B
1.47E-01	2.08E-01	No	Cluster B
9.86E-02	1.55E-01	No	Cluster B
1.14E-01	1.70E-01	No	Cluster B
7.99E-01	6.46E-01	No	Cluster B
4.53E-02	8.55E-02	No	Cluster B
5.05E-01	4.33E-01	No	Cluster B
1.63E-01	2.19E-01	No	Cluster B
2.54E-01	2.56E-01	No	Cluster B
4.63E-03	1.64E-02	No	Cluster C
2.07E-01	2.40E-01	No	Cluster C
5.61E-01	4.66E-01	No	Cluster C
8.60E-02	1.43E-01	No	Cluster C
1.28E-02	2.70E-02	No	Cluster C
2.06E-01	2.40E-01	No	Cluster C
3.31E-01	3.02E-01	No	Cluster C
7.39E-03	2.23E-02	No	Cluster C
1.34E-02	2.70E-02	No	Cluster D
2.65E-01	2.56E-01	No	Cluster D
7.91E-02	1.40E-01	No	Cluster D
8.66E-03	2.23E-02	No	Cluster D
3.47E-01	3.06E-01	No	Cluster D
2.44E-01	2.56E-01	No	Cluster D
2.72E-01	2.56E-01	No	Cluster D
2.13E-01	2.40E-01	No	Cluster D
