# Supplementary figures and images for: Dysregulation of the leukocyte signaling landscape during acute COVID-19
Source: PLoS One. 2022 Apr 14;17(4):e0264979. doi: 10.1371/journal.pone.0264979 (PMC9009616; doi:10.1371/journal.pone.0264979)

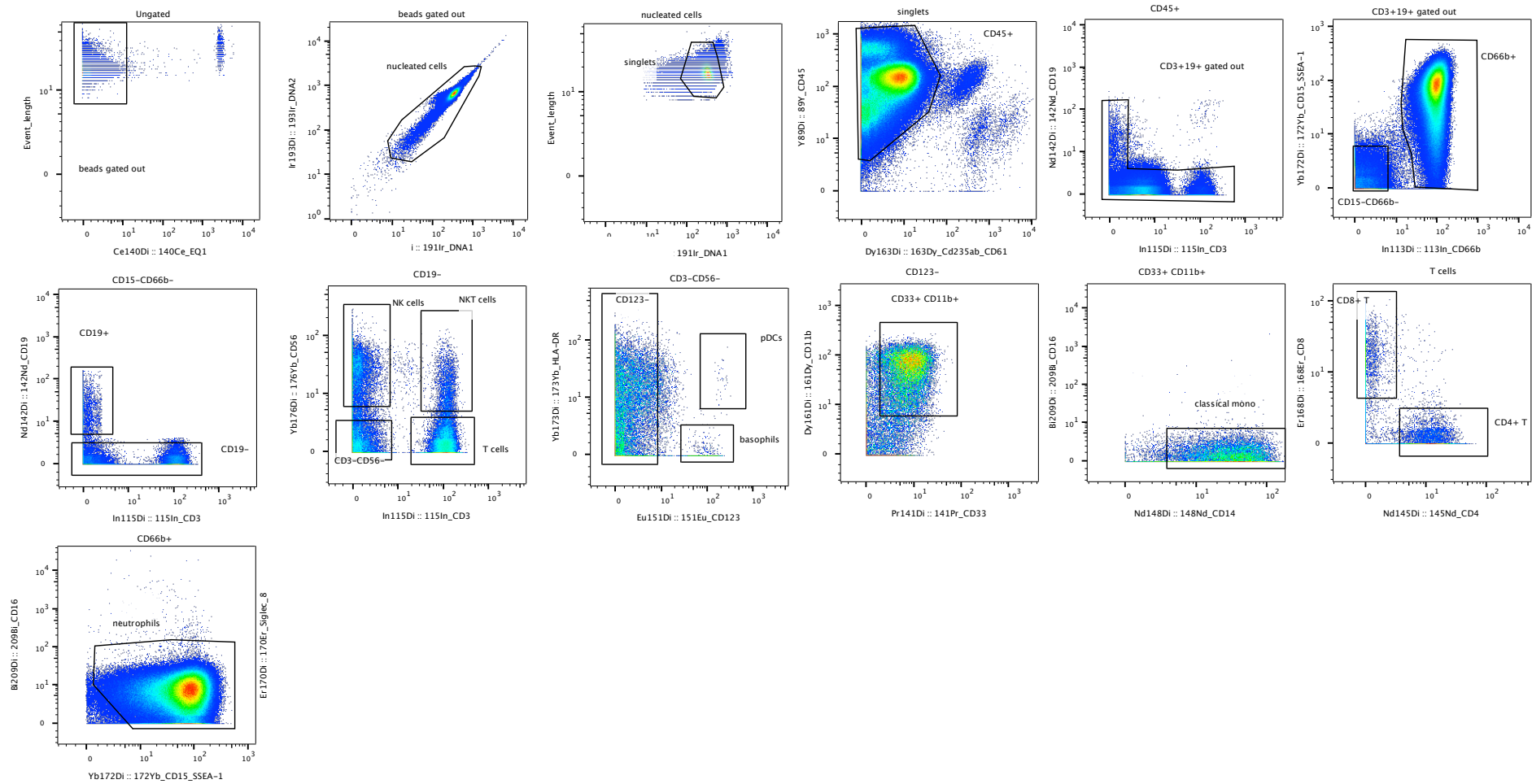

Supplement: S1 Fig — (PDF) [file pone.0264979.s006.pdf]

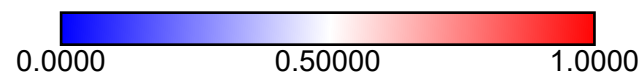

Healthy Donor  
Moderate COVID19  
Severe COVID19

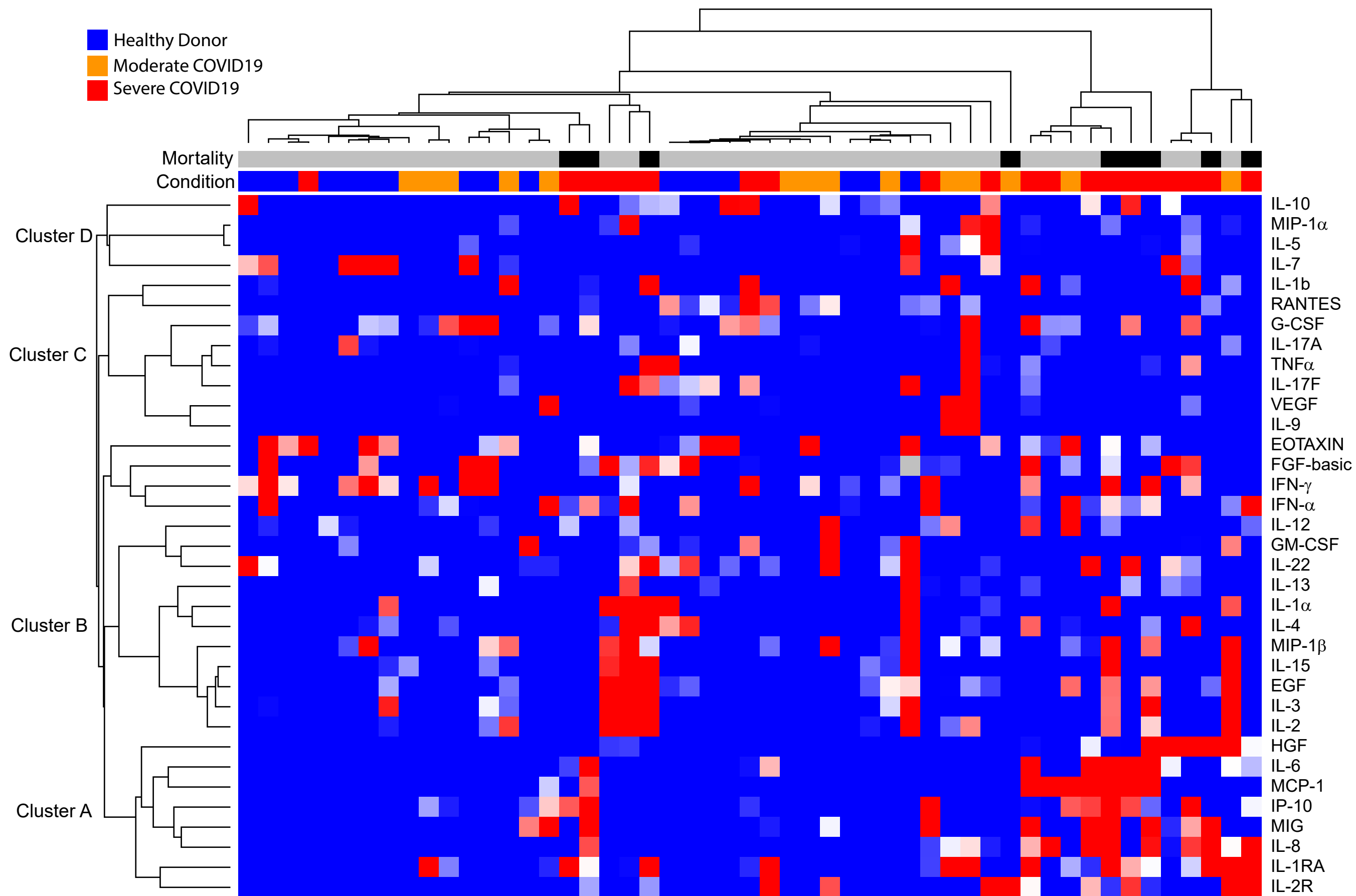

Supplement: S2 Fig — (PDF) [file pone.0264979.s007.pdf]
